# Body mass index and health-related lifestyle behaviors among university students in Saudi Arabia: a single center cross-sectional study

**DOI:** 10.3389/fpubh.2026.1900451

**Published:** 2026-10-14

**Authors:** Nora Alafif, Shabana Tharkar, Rinas Alharbi, Hadeel Althobaiti, Rawa Alamry, Reema Al-harbi, Waad Alfawaz

**Affiliations:** Department of Community Health Science, College of Applied Medical Sciences, King Saud University, Riyadh, Saudi Arabia

**Keywords:** body mass index, dietary behavior, physical activity, Saudi Arabia, university students

## Abstract

**Background:**

Overweight and obesity are important public-health concerns among young adults. University students experience changes in dietary, physical-activity, sleep, and other health behaviors, but evidence from Saudi university populations remains heterogeneous.

**Objective:**

To explore associations between BMI and selected dietary, physical-activity, sleep, and sociodemographic characteristics among university students.

**Methods:**

This cross-sectional study analyzed electronic questionnaire responses collected from January to April 2025 on a sample of 196 students. Weight and height were self-reported and BMI was recalculated as weight (kg)/height^2^ (m^2^). BMI was categorized as underweight (<18.5), normal (18.5 to <24.9), overweight (25 to <30), or obesity (≥30 kg/m^2^). Associations were examined using contingency-table analyses and limited exploratory multivariable linear and logistic regression models.

**Results:**

Among 196 analyzed students, 17 (8.7%) were underweight, 106 (54.1%) had normal BMI, 38 (19.4%) were overweight, and 35 (17.9%) had obesity; thus, 73 (37.2%) had overweight or obesity. Associations of BMI category with lifestyle were not statistically significant. Electronic-device use before bed showed a sparse-cell association (*χ*^2^=23.438, df = 9, Monte Carlo *p* = 0.010) that should be considered exploratory. In adjusted linear regression, male sex (*β* = 5.26 kg/m^2^, 95% CI 2.36–8.16, *p* < 0.001), and students aged >23 years had higher BMI (*β* = 2.86, 95% CI 0.44–5.29, *p* = 0.021). In adjusted logistic regression, male sex (OR = 2.71, 95% CI 1.19–6.19, *p* = 0.018) and age >23 years (OR = 3.15, 95% CI 1.17–8.43, *p* = 0.023) were associated with overweight/obesity.

**Conclusion:**

Overweight and obesity were common. Gender and age showed associations with BMI status, whereas evidence for independent associations of the selected lifestyle measures was limited and imprecise. The cross-sectional design and self-reported measurements preclude causal inference

## 1 Introduction

Body mass index (BMI) is a widely recognized anthropometric measurement utilized as a screening instrument to estimate an individual’s body fat in relation to their height and weight. It is calculated by dividing a person’s weight in kilograms by their height in square meters (kg/m^2^) (1). BMI provides a straightforward numerical value that categorizes individuals into various weight-to-height categories, with elevated BMI frequently associated with the risk of non-communicable diseases such as diabetes and cardiovascular conditions (2). For adults, commonly used categories are underweight (<18.5 kg/m^2^), normal weight (18.5 to <24.9 kg/m^2^), overweight (25 to <30 kg/m^2^), and obesity (≥30 kg/m^2^). Lifestyle behaviors significantly influence BMI. Research suggests that a sedentary lifestyle and unhealthy dietary habits are primary drivers of high BMI, resulting in overweight and obesity (3, 4). Previously, overweight and obesity were perceived as issues primarily affecting high-income nations; however, in recent decades, they have evolved into a global health concern, increasingly prevalent across all populations (5, 6). The phenomenon of nutrition transition, characterized by high-calorie food that is easily accessible and affordable, in combination with sedentarism due to urbanization, has led to catastrophic effects on population health (7, 8). In Saudi Arabia, overweight and obesity have emerged as critical public health challenges, with recent 2024 data indicating that 23.1% of adults older than 15 years are obese and 45.1% are overweight (9). This trend is primarily attributed to rapid urbanization, which has led to a shift towards sedentary lifestyles and a diet increasingly dominated by ultra-processed fast foods and sugar-sweetened beverages (9).

The lifestyle habits of university students have a significant impact on their BMI. The shift to university life often represents a period of increased risk for overweight and obesity, rendering students more susceptible. Research shows that insufficient physical activity and unhealthy dietary practices, including the frequent intake of calorie-dense fast food, sugary drinks, and a low consumption of fruits and vegetables, are key contributors to the rise in BMI within this demographic (10, 11). A sedentary lifestyle, exacerbated by prolonged screen time for both academic and recreational purposes, further heightens this risk (12). In addition to diet and physical activity, poor sleep quality and academic pressure also significantly contribute to the increase in BMI, impacting metabolic health and eating habits that may result in weight gain. This highlights the necessity for comprehensive lifestyle strategies aimed at sustaining a healthy BMI throughout the university years (13). In Saudi Arabia, research in this area is still limited and frequently concentrates on individual lifestyle factors rather than their collective impacts. Addressing these deficiencies is crucial for comprehending the intricate factors that affect BMI and for formulating effective public health policies aimed at students. The present study therefore examined associations between self-reported anthropometric BMI and selected dietary, physical-activity, sleep, and sociodemographic variables among students at a large University. Because the design was cross-sectional, the study was intended to identify associations rather than establish causal effects The primary outcome was continuous BMI, with a secondary categorical analysis comparing normal BMI with overweight/obesity.

## 2 Methods

### 2.1 Study design and participants

A cross-sectional study was conducted among students enrolled at King Saud University, Riyadh, Saudi Arabia, during January–April 2025. The study examined the association between lifestyle behaviors, including sleep quality, physical activity, and dietary habits, on body mass index among university students. The research involved 196 students aged 18–34, from various colleges within a large university.

### 2.2 Eligibility criteria

Eligibility criteria were students aged 18–34 years who provided informed consent. The original protocol excluded individuals who did not consent, were pregnant or breastfeeding women, and individuals older than 34 years.

### 2.3 Sample size estimation

The minimum required sample size for this study was calculated using Cochran’s formula for cross-sectional designs: *n* = *Z*^2^ *p*(1−*p*)/*d*^2^. The expected prevalence (*P*) was set at 14.6% (0.146), referencing the documented prevalence of obesity among Saudi university students in previous regional literature (14), setting the standard normal deviation at 1.96 (for a 95% confidence interval) and the absolute margin of error (*d*) at 5% (0.05), the initial calculation yielded an optimal sample size of approximately 195 participants.

### 2.4 Sampling technique

A stratified random sampling method was utilized to select the target population from each college as strata and years of study as substrata. Following selection of each year, random sampling technique was applied to select the students. The selected students were explained about the purpose of study and were informed about voluntary participation and right to withdraw anytime during the procedure. After getting the informed consent, the link containing electronic questionnaire was shared with the students via the email ID or social media platform. Data collection was done using the Google Docs in the SurveyMonkey Platform. Anonymity was maintained throughout the data collection procedure to ensure confidentiality.

### 2.5 Measures

#### 2.5.1 Age-group definitions

Age was analyzed using the non-overlapping categories 18–20, 21–23, and >23 years.

#### 2.5.2 Anthropometry

Participants self-reported the most recent measured weight in kilograms and height in centimeters. BMI was recalculated from these values using BMI = weight (kg)/height^2^ (m^2^). BMI categories were defined as underweight (<18.5 kg/m^2^), normal (18.5 to <24.9 kg/m^2^), overweight (25 to <30 kg/m^2^), and obesity (≥30 kg/m^2^).

#### 2.5.3 Sleep

Sleep duration and a single subjective sleep-quality item with responses “Very bad,” “Bad,” “Good,” and “Very good,” were recorded. The questionnaire did not include responses needed to reproduce a Pittsburgh Sleep Quality Index (PSQI) global score or its seven component scores. No global PSQI cutoff was applied.

#### 2.5.4 Physical activity

The survey tool included categorical exercise frequency, activity duration, activity type, activity goal, and several Likert items about beliefs concerning physical activity. It did not include the complete item-level International Physical Activity Questionnaire (IPAQ) data needed to calculate MET-minutes/week or classify participants as low, moderate, or high activity according to IPAQ scoring rules was not included to calculate the score.

#### 2.5.5 Dietary behavior

Dietary variables were assessed using frequency questions concerning meals per day, breakfast, fruit/vegetable intake, fried/fast-food intake, sweets/sugar-sweetened drinks, water intake, and one self-perception item asking whether eating habits were considered healthy. “Healthy eating” refers specifically to a participant answering “yes” to the question “Are your eating habits considered healthy?”; “unhealthy eating” refers to “no.” The terms ‘healthy’ and ‘unhealthy’ eating refer only to the respondent’s answer to the exact self-rating question and do not represent a validated dietary-quality score. Protein sources included eggs, meat, chicken, and any form of protein supplements.

### 2.6 Survey tool

The questionnaire incorporated several validated tools, such as the Pittsburgh Sleep Quality Index (PSQI) (14), which was used to evaluate sleep quality over the preceding month; the Food Frequency Questionnaire (FFQ) (15), which assessed eating habits, including meal timing, fruit and vegetable intake, and caffeine consumption; the International Physical Activity Questionnaire (16) (IPAQ), which measured physical activity levels in terms of frequency, duration, and intensity; and self-reported anthropometric data, including weight and height, which were used to calculate BMI (kg/m^2^).

The original versions of IPAC and PQSI questionnaires were pilot tested on 20 students to assess the reliability of the questionnaire. After the pilot on 20 students, it was realized that scores of both IPAC and PQSI tools were incomplete with complete missing data. Hence the scores were removed from the final questionnaire. An electronic version of the questionnaire was created in GoogleDocs for data collection.

### 2.7 Variables

The primary outcome was BMI, calculated as weight in kilograms divided by height in meters squared. BMI categories were underweight (<18.5 kg/m^2^), normal weight (18.5 to <24.9 kg/m^2^), overweight (25 to <30 kg/m^2^), and obesity (≥30 kg/m^2^). For the secondary binary analysis, participants with BMI 18.5 to <25 kg/m^2^ were the reference normal-BMI group and those with BMI ≥ 25 kg/m^2^ were classified as overweight/obesity; underweight participants were excluded from this binary comparison. The independent variables included sleep quality, physical activity, and dietary habits, each evaluated through a designated questionnaire. Additionally, covarites were explored for their possible on association with BMI, encompassing demographic factors such as sex, age, type of college, and academic degree.

### 2.8 Statistical analysis

Descriptive statistics are presented as frequencies and percentages for categorical variables and mean ± standard deviation for continuous variables. BMI was treated as a continuous primary outcome in multivariable analysis and as a four-category outcome for descriptive and exploratory association analyses. A secondary binary outcome was defined as overweight/obesity (BMI ≥ 25 kg/m^2^) versus normal BMI (18.5 to <24.9 kg/m^2^); underweight participants were excluded from this binary comparison rather than combined with normal-weight participants.

Associations between categorical variables and four-category BMI status were evaluated with Pearson chi-square statistics. Because several contingency tables contained expected counts below five, Monte Carlo exact *p*-values based on 20,000 random tables conditional on the observed margins were used for sparse tables. These *p*-values are reported as exploratory association tests rather than as evidence of causation.

The primary multivariable analysis used ordinary least-squares regression with continuous BMI as the dependent variable and sex, age group, meal frequency, perceived healthy eating, exercise frequency, and sleep duration as predictors. Because residual diagnostics indicated non-normality, heteroscedasticity-robust HC3 standard errors were used.

The secondary multivariable analysis used logistic regression for overweight/obesity versus normal BMI. Predictors were sex, age group, meal-frequency category, and perceived healthy eating. Adjusted odds ratios (ORs) and 95% confidence intervals (CIs) are reported. The meal-frequency contrast with >3 meals/day had a wide CI and is interpreted as imprecise. Predictors were not selected on the basis of univariable *p*-values.

Because numerous exploratory lifestyle associations were examined, *p*-values were not formally adjusted for multiplicity. The lifestyle cross-tabulations are therefore considered hypothesis-generating, and interpretation emphasizes effect magnitude, uncertainty, sparse cells, and consistency rather than isolated threshold crossing at *p* < 0.05.

### 2.9 Bias

Potential selection bias may arise from single-university recruitment and possible differences between participants and the underlying student population. Volunteer/nonresponse bias is possible because participation was voluntary and the number invited and response rate were not available. Information bias may result from self-reported height, weight, diet, sleep, and physical activity. Recall bias is relevant to retrospective reports of behaviors. Social-desirability bias may cause participants to underreport behaviors perceived as unhealthy. Single-center sampling limits external validity. These risks were reduced, where possible, by standardized electronic questions and confidential participation, but cannot be eliminated.

### 2.10 Ethical considerations

Ethical approval for this study was obtained from the Institutional Review Board, reference number 4/67/217615 on 13th February 2025. The research was conducted in accordance with the Declaration of Helsinki, and the STROBE guidelines for cross-sectional studies were adhered to at every stage. Anonymity was preserved to enhance confidentiality, and informed consent was secured from all participants.

## 3 Results

### 3.1 Participant characteristics

The final analytic sample comprised 196 students. Mean BMI was 24.87 ± 6.30 kg/m^2^ (median 23.61; range 14.13–47.31). There were 17 underweight participants (8.7%), 106 with normal BMI (54.1%), 38 were overweight (19.4%), and 35 with obesity (17.9%). Thus, 73 participants (37.2%) had overweight or obesity.

The other sociodemographic details are presented in Table 1.

**Table 1:** Characteristics of eligible study participants (*n* = 196).

Characteristics of study population	*N* (%) or mean ± SD
Age, years	
18–20 years	76 (38.8)
21–23 years	91 (46.4)
Over 23 years	29 (14.8)
Sex	
Female	155 (79.1)
Male	41 (20.9)
College	
Health Colleges	103 (52.6)
College of Business Administration	43 (21.9)
Scientific Colleges	25 (12.8)
Humanities Colleges	25 (12.8)
BMI category	
Normal	106 (54.1)
Overweight	38 (19.4)
Obesity	35 (17.9)
Underweight	17 (8.7)
Weight, kg	65.7 ± 21.1
Height, cm	161.9 ± 9.4
BMI, kg/m^2^	24.87 ± 6.30

Characteristics of the final analytic sample (*N* = 196). Percentages are based on the full analytic sample. BMI categories follow adult thresholds: underweight <18.5, normal 18.5 to <24.9, overweight 25 to <30, and obesity ≥30 kg/m^2^.

### 3.2 Sociodemographic characteristics and BMI category

Table 2 presents the distribution of BMI according to sociodemographic characteristics. The distribution of BMI categories did not differ significantly across age groups (*χ*^2^ = 6.575, df = 6, Pearson *p* = 0.362; Monte Carlo *p* = 0.364). Among participants aged 18–20 years, 59.2% had normal BMI, compared with 51.6% of those aged 21–23 years and 48.3% of those aged >23 years. The proportion classified as overweight increased from 13.2% among participants aged 18–20 years to 31.0% among those aged >23 years; however, the overall association was not statistically significant. In contrast, BMI category was significantly associated with sex (*χ*^2^ = 17.290, df = 3, Pearson *p* < 0.001; Monte Carlo *p* = 0.001). Among women, 56.1% had normal BMI, 21.3% were overweight, 12.3% had obesity, and 10.3% were underweight. Among men, 46.3% had normal BMI, 12.2% were overweight, 39.0% had obesity, and 2.4% were underweight.

**Table 2:** BMI status according to sociodemographic characteristics.

Characteristics	Category	Under weight	Normal	Overweight	Obese	*χ* ^2^	df	Pearson *p*	Monte Carlo *p*
Age, years	18–20	9 (11.8)	45 (59.2)	10 (13.2)	12 (15.8)	6.57	6	0.362	0.364
	21–23	7 (7.7)	47 (51.6)	19 (20.9)	18 (19.8)				
	>23	1 (3.4)	14 (48.3)	9 (31)	5 (17.2)				
Sex	Female	16 (10.3)	87 (56.1)	33 (21.3)	19 (12.3)	17.29	3	<0.001	0.001
	Male	1 (2.4)	19 (46.3)	5 (12.2)	16 (39)				
College	College of Business Administration	1 (2.3)	23 (53.5)	6 (14)	13 (30.2)	9.79	9	0.368	0.354
	Health Colleges	12 (11.7)	53 (51.5)	23 (22.3)	15 (14.6)				
	Humanities Colleges	2 (8)	14 (56)	5 (20)	4 (16)				
	Scientific Colleges	2 (8)	16 (64)	4 (16)	3 (12)				

BMI categories follow adult thresholds: underweight <18.5, normal 18.5 to <24.9, overweight 25 to <30, and obesity ≥30 kg/m^2^.

### 3.3 Dietary, sleep, and physical-activity characteristics by BMI category

Table 3 illustrates the BMI categories according to lifestyle characteristics. The distribution of BMI categories did not differ significantly according to meal frequency (*χ*^2^ = 10.870, df = 9, Pearson *p* = 0.285; Monte Carlo *p* = 0.281). Participants reporting more than three meals per day showed a comparatively higher proportion of overweight (42.9%) and obesity (28.6%). However, these descriptive differences did not constitute statistically significant evidence of an overall association. Similarly, perceived healthy eating was not significantly associated with BMI category (*χ*^2^ = 2.093, df = 3, Pearson *p* = 0.553; Monte Carlo *p* = 0.553). Breakfast frequency (*χ*^2^ = 9.108, df = 9, Pearson *p* = 0.427; Monte Carlo *p* = 0.421) and water intake (*χ*^2^ = 9.641, df = 9, Pearson *p* = 0.380; Monte Carlo *p* = 0.386) were also not significantly associated with BMI category.

**Table 3:** Associations between lifestyle behaviors and BMI category (*N* = 196).

Variable	Category	Underweight	Normal	Overweight	Obesity	*χ* ^2^	df	Pearson *p*	Monte Carlo *p*
		*n* (%)	*n* (%)	*n* (%)	*n* (%)				
Meal frequency	One meal	2 (10.5)	11 (57.9)	2 (10.5)	4 (21.1)	10.870	9	0.285	0.281
	Two meals	10 (9.3)	57 (52.8)	22 (20.4)	19 (17.6)				
	Three meals	4 (7.3)	35 (63.6)	8 (14.5)	8 (14.5)				
	>3 meals	1 (7.1)	3 (21.4)	6 (42.9)	4 (28.6)				
Perceived healthy eating	No	11 (8.3)	68 (51.5)	26 (19.7)	27 (20.5)	2.093	3	0.553	0.553
	Yes	6 (9.4)	38 (59.4)	12 (18.8)	8 (12.5)				
Breakfast	Always	4 (8.7)	26 (56.5)	11 (23.9)	5 (10.9)	9.108	9	0.427	0.421
	Never	–	5 (55.6)	1 (11.1)	3 (33.3)				
	Probably	11 (13.6)	42 (51.9)	14 (17.3)	14 (17.3)				
	Rarely	2 (3.3)	33 (55.0)	12 (20.0)	13 (21.7)				
Water intake	<1 L	7 (13.7)	29 (56.9)	9 (17.6)	6 (11.8)	9.641	9	0.380	0.386
	1–1.5 L	8 (9.3)	46 (53.5)	14 (16.3)	18 (20.9)				
	1.6–2 L	1 (2.9)	21 (61.8)	7 (20.6)	5 (14.7)				
	>2 L	1 (4.0)	10 (40.0)	8 (32.0)	6 (24.0)				
Sleep duration	<4 h	–	5 (62.5)	–	3 (37.5)	10.341	9	0.324	0.317
	4–6 h	5 (7.9)	29 (46.0)	13 (20.6)	16 (25.4)				
	7–8 h	8 (8.8)	54 (59.3)	19 (20.9)	10 (11.0)				
	>8 h	4 (11.8)	18 (52.9)	6 (17.6)	6 (17.6)				
Sleep quality	Very bad	3 (15.8)	13 (68.4)	3 (15.8)	0 (0.0)	10.429	9	0.317	0.321
	Bad	4 (6.9)	35 (60.3)	8 (13.8)	11 (19.0)				
	Good	9 (10)	43 (47.8)	19 (21.1)	19 (21.1)				
	Very good	1 (3.4)	15 (51.7)	8 (27.6)	5 (17.2)				
Electronic devices before bed	Never	–	1 (50.0)	–	1 (50.0)	23.438	9	0.005	0.010
	Rarely	–	6 (66.7)	2 (22.2)	1 (11.1)				
	Probably	–	34 (63.0)	4 (7.4)	16 (29.6)				
	Always	17 (13)	65 (49.6)	32 (24.4)	17 (13.0)				
Fruit/vegetable frequency	Never	2 (7.4)	17 (63.0)	4 (14.8)	4 (14.8)	8.482	9	0.486	0.496
	1–2 times	10 (11.8)	40 (47.1)	18 (21.2)	17 (20.0)				
	3–4 times	4 (8.2)	27 (55.1)	7 (14.3)	11 (22.4)				
	Daily	1 (2.9)	22 (62.9)	9 (25.7)	3 (8.6)				
Fast-food frequency	Never	1 (5.0)	13 (65.0)	4 (20.0)	2 (10.0)	8.454	9	0.489	0.497
	1–2 times	9 (8.5)	54 (50.9)	26 (24.5)	17 (16.0)				
	3–4 times	6 (10.5)	32 (56.1)	5 (8.8)	14 (24.6)				
	Daily	1 (7.7)	7 (53.8)	3 (23.1)	2 (15.4)				
Sweets/sugary drinks frequency	Never	2 (16.7)	4 (33.3)	3 (25.0)	3 (25.0)	10.595	9	0.305	0.307
	1–2 times	9 (9.6)	55 (58.5)	18 (19.1)	12 (12.8)				
	3–4 times	1 (2.0)	26 (52.0)	9 (18.0)	14 (28.0)				
	Daily	5 (12.5)	21 (52.5)	8 (20.0)	6 (15.0)				
Exercise frequency	Do not get physically active	4 (11.8)	21 (61.8)	5 (14.7)	4 (11.8)	5.952	12	0.919	0.926
	Rarely	6 (9.5)	32 (50.8)	12 (19.0)	13 (20.6)				
	1–2 times	3 (6.2)	24 (50.0)	12 (25.0)	9 (18.8)				
	3–4 times	2 (6.7)	15 (50.0)	7 (23.3)	6 (20.0)				
	>4 times	2 (9.5)	14 (66.7)	2 (9.5)	3 (14.3)				

Values are presented as *n* (%), where percentages are calculated within each response category across the four BMI categories. Pearson *χ*^2^ tests were used for omnibus associations. Because several contingency tables contained sparse cells, conditional Monte Carlo exact *p*-values (20,000 replicates) are also reported. BMI categories were defined as underweight (<18.5 kg/m^2^), normal (18.5 to <24.9 kg/m^2^), overweight (25 to <30 kg/m^2^), and obesity (≥30 kg/m^2^). The electronic-device association should be interpreted cautiously because of very small cell counts.

No statistically significant association was observed between sleep duration and BMI category. For sleep duration, obesity was observed in 37.5% of participants reporting <4 h of sleep, compared with 25.4% among those reporting 4–6 h, 11.0% among those reporting 7–8 h, and 17.6% among those reporting >8 h; the small number of participants in the <4-h category warrants cautious interpretation.

The four-category distribution of BMI differed significantly according to electronic-device use before bedtime (*χ*^2^ = 23.438, df = 9, Pearson *p* = 0.005; Monte Carlo *p* = 0.010). In particular, 29.6% of participants who reported “probably” using electronic devices before bed were classified as having obesity, compared with 13.0% among those reporting “always.” However, several categories contained very small numbers, including only two participants reporting “never” and nine reporting “rarely”; therefore, this finding should be considered exploratory.

### 3.4 Multivariable analysis of continuous BMI

Table 4 shows multivariable linear regression model that examined the independent associations of sex, age group, meal frequency, perceived healthy eating, exercise frequency, and sleep duration with continuous BMI. The model explained approximately 16.9% of the variance in BMI (*R*^2^ = 0.169; adjusted *R*^2^ = 0.138).

**Table 4:** Adjusted associations with continuous BMI.

Predictor	*β* (kg/m^2^)	95% CI	*p*
Male vs. female	5.26	2.36 to 8.16	<0.001
Age 21–23 vs. 18–20 years	1.58	−0.26 to 3.43	0.092
Age >23 vs. 18–20 years	2.86	0.44 to 5.29	0.021
Each one-category increase in meal frequency	0.83	−0.36 to 2.02	0.174
Perceived healthy eating: yes vs. no	−2.23	−4.15 to −0.31	0.023
Each one-category increase in exercise frequency	0.03	−0.75 to 0.81	0.937
Each one-category increase in sleep duration	−0.99	−2.11 to 0.12	0.080

Multivariable linear regression of continuous BMI (*N* = 196). Model *R*^2^ = 0.169; adjusted *R*^2^ = 0.138. HC3 heteroscedasticity-robust standard errors were used. Reference categories were female sex and age 18–20 years. Meal frequency, exercise frequency, and sleep duration were entered as ordered categories.

Sex showed the strongest association with BMI in the adjusted model. Men had, on average, a BMI 5.26 kg/m^2^ higher than women (*β* = 5.26, 95% CI 2.36–8.16, *p* < 0.001). Participants aged >23 years also had higher BMI than those aged 18–20 years (*β* = 2.86, 95% CI 0.44–5.29, *p* = 0.021), whereas the difference between participants aged 21–23 years and those aged 18–20 years was not statistically significant (*β* = 1.58, 95% CI −0.26 to 3.43, *p* = 0.092).

Perceived healthy eating was inversely associated with BMI in the adjusted model; participants who reported that they perceived their eating as healthy had a BMI approximately 2.23 kg/m^2^ lower than those who did not (*β* = −2.23, 95% CI −4.15 to −0.31, *p* = 0.023). Meal frequency was not significantly associated with BMI (*β* = 0.83 per category increase, 95% CI −0.36 to 2.02, *p* = 0.174). Neither exercise frequency (*β* = 0.03, 95% CI −0.75 to 0.81, *p* = 0.937) nor sleep-duration category (*β* = −0.99, 95% CI −2.11 to 0.12, *p* = 0.080) showed statistically significant adjusted associations with BMI.

### 3.5 Multivariable analysis of overweight/obesity

Table 5 presents the adjusted associations with overweight and obesity. A secondary logistic regression analysis was conducted among 179 participants with normal BMI or overweight/obesity, excluding participants classified as underweight. The outcome was overweight/obesity (BMI ≥ 25 kg/m^2^) versus normal BMI (18.5 to <25 kg/m^2^). The model included sex, age group, meal-frequency category, and perceived healthy eating Overall, the adjusted analyses consistently identified sex and, to a lesser extent, older age as the variables most clearly associated with BMI-related outcomes in this sample. The findings for meal frequency and perceived healthy eating were less precise in the categorical outcome model, as reflected by the wide confidence intervals.

**Table 5:** Adjusted associations with overweight/obesity.

Predictor/contrast	Adjusted OR	95% CI	*p*
Meals: more than three meals	5.42	0.97–30.20	0.054
Meals: three meals	0.59	0.17–2.06	0.405
Meals: two meals	0.99	0.32–3.06	0.987
Male vs. female	2.71	1.19–6.19	0.018
Age 21–23 vs. 18–20 years	1.96	0.93–4.11	0.076
Age >23 vs. 18–20 years	3.15	1.17–8.43	0.023
Perceived healthy eating: yes vs. no	0.62	0.30–1.28	0.198

Multivariable logistic regression for overweight/obesity versus normal BMI (*N* = 179; underweight excluded). Model likelihood-ratio *p* = 0.005; pseudo-*R*^2^ = 0.083. Reference categories: female sex, age 18–20 years, one meal/day, and perceived unhealthy eating (no). ORs are adjusted estimates with 95% CIs.

Men had significantly higher odds of overweight/obesity than women after adjustment (adjusted OR = 2.71, 95% CI 1.19–6.19, *p* = 0.018). Participants aged >23 years also had higher odds of overweight/obesity compared with those aged 18–20 years (adjusted OR = 3.15, 95% CI 1.17–8.43, *p* = 0.023), whereas the association for participants aged 21–23 years was not statistically significant (adjusted OR = 1.96, 95% CI 0.93–4.11, *p* = 0.076).

Compared with participants reporting one meal per day, those reporting more than three meals per day had higher estimated odds of overweight/obesity (adjusted OR = 5.42, 95% CI 0.97–30.20, *p* = 0.054); however, the confidence interval included the null value and the association did not reach conventional statistical significance. The corresponding estimates for three meals (adjusted OR = 0.59, 95% CI 0.17–2.06, *p* = 0.405) and two meals (adjusted OR = 0.99, 95% CI 0.32–3.06, *p* = 0.987) were not statistically significant. Perceived healthy eating was also not significantly associated with overweight/obesity after adjustment (adjusted OR = 0.62, 95% CI 0.30–1.28, *p* = 0.198).

## 4 Discussion

### 4.1 Principal findings

This study examined associations between BMI and selected dietary, physical activity, and sleep-related behaviors among university students in Saudi Arabia. Among 196 participants, the mean BMI was 24.87 ± 6.30 kg/m^2^, and 37.2% were classified as overweight or obese. The principal finding was that sex and age were the most consistent correlates of BMI. Male sex and age >23 years were independently associated with higher continuous BMI and greater odds of overweight/obesity. Students who perceived their eating habits as healthy had lower continuous BMI. In contrast, most individual dietary, physical activity, and sleep variables were not independently associated with BMI after adjustment.

The prevalence of overweight/obesity observed here is broadly consistent with the substantial burden reported among university students in Saudi Arabia and other Gulf countries. A recent study at the University of Ha’il reported overweight and obesity prevalences of 26.4 and 17.1%, respectively, giving a combined prevalence of approximately 43.5% (17). Similarly, students at Jazan University had reported prevalences of 20.4% for overweight and 14.9% for obesity (18). Our estimate of 37.2% therefore falls within the range reported in Saudi university populations, although differences in sampling, sex composition, age distribution, and measurement methods limit direct comparisons.

Comparable concerns have been reported elsewhere in the Gulf. Studies among university students in Oman and Kuwait have documented substantial levels of overweight and obesity (19, 20), while more recent evidence from Oman continues to identify excess weight as an important health concern among college students (21). These regional findings occur within a broader global obesity epidemic. The Global Burden of Disease 2021 analysis estimated that approximately 45% of adults aged ≥25 years worldwide were living with overweight or obesity in 2021 (22). A global systematic review and meta-analysis of medical students reported pooled prevalences of approximately 18% for overweight and 9% for obesity, with higher prevalence generally observed among males (23). Although the populations and methodologies differ, these findings reinforce the relevance of excess weight during the university years.

### 4.2 Sex and age differences in BMI

Sex was the strongest and most consistent correlate in the present study. Male students had a higher adjusted BMI than females (*β* = 5.26 kg/m^2^, 95% CI 2.36–8.16) and higher adjusted odds of overweight/obesity (OR = 2.71, 95% CI 1.19–6.19). BMI distribution also differed significantly by sex.

This finding is consistent with studies from Kuwait and Oman reporting greater overweight/obesity among male university students (19, 20). It also agrees with the global medical-student meta-analysis, which generally found a higher prevalence of excess weight among males (23). However, findings are not uniform. Recent Saudi research has demonstrated substantial excess weight among female students as well, illustrating that sex differences may vary between institutions and populations (17).

Potential explanations include differences in dietary practices, physical activity, sedentary behavior, body composition, and sociocultural factors. However, these mechanisms were not directly assessed in the present study. BMI also cannot distinguish lean mass from adipose tissue; therefore, the higher BMI observed among males should not automatically be interpreted as proportionally greater adiposity.

Older age was also associated with BMI. Students aged >23 years had higher adjusted BMI than those aged 18–20 years (*β* = 2.86 kg/m^2^, 95% CI 0.44–5.29) and approximately threefold greater odds of overweight/obesity (OR = 3.15, 95% CI 1.17–8.43). The 21–23-year group showed the same direction of association but did not reach statistical significance. Evidence from other student populations is mixed; for example, age was not significantly associated with overweight/obesity in the recent University of Ha’il study (17), whereas increasing age has been associated with excess weight in younger populations in Qatar (24). Longitudinal studies are needed to determine whether BMI increases during university and which behavioral changes accompany such trajectories.

### 4.3 Dietary behaviors

Most dietary variables—including meal frequency, breakfast consumption, water intake, fruit and vegetable consumption, fast-food consumption, and sweets—were not significantly associated with BMI category. Meal frequency was also not independently associated with continuous BMI.

These findings differ from some Saudi studies reporting relationships between BMI and dietary practices. Studies among students at Jazan University and the University of Ha’il identified associations between excess weight and selected dietary behaviors (17, 18). Differences may reflect variations in populations and, importantly, measurement methods.

The present questionnaire assessed dietary behaviors primarily through frequency categories and did not quantify portion size, total energy intake, nutrient composition, or overall dietary patterns. Thus, students reporting similar frequencies may have had substantially different dietary exposures. The absence of significant associations should therefore not be interpreted as evidence that these dietary behaviors are unrelated to body weight.

Students who perceived their eating habits as healthy had lower adjusted BMI (*β* = −2.23 kg/m^2^, 95% CI −4.15 to −0.31). However, this was a subjective single-item measure rather than an objective assessment of diet quality. Reverse causation is also possible, as students with lower BMI may be more likely to perceive their eating habits positively. This finding should therefore be interpreted cautiously.

### 4.4 Physical activity and sleep

Exercise frequency was not significantly associated with BMI (*β* = 0.03 kg/m^2^ per category increase, 95% CI −0.75 to 0.81). This contrasts with some regional studies reporting relationships between physical activity or sedentary behaviors and weight status (17, 19).

Self-reported activity may also be affected by recall and social-desirability bias. Reverse causation is possible because students with higher BMI may increase exercise in response to concerns about their weight. These factors may weaken associations in cross-sectional analyses.

Sleep duration and sleep quality were likewise not significant independent correlates of BMI. This differs from some recent Saudi evidence. A large study among university students in Riyadh reported an association between BMI and poor sleep quality using the Pittsburgh Sleep Quality Index (PSQI) (25). International research has frequently linked short or poor-quality sleep with obesity, but the strength and direction of these relationships vary between populations and may depend on age, sex, physical activity, diet, and other confounders.

### 4.5 Electronic-device use

Electronic-device use before bedtime was significantly associated with the four-category BMI outcome. However, this result should be regarded as exploratory because several response categories contained very few participants. Although a Monte Carlo procedure was used to address sparse contingency-table inference, estimates remain unstable.

The finding is potentially consistent with broader research linking screen exposure with sedentary behavior, sleep disruption, and obesity-related outcomes. Nevertheless, our study did not quantify total screen time, sedentary duration, or the timing and purpose of device use. Therefore, no causal or mechanistic conclusion can be drawn. Given the sparse cells and the number of lifestyle comparisons performed, replication in larger samples is necessary.

### 4.6 Interpretation of lifestyle findings

The predominance of null associations differs from some Saudi studies that identified relationships between excess weight and multiple lifestyle behaviors. However, this discrepancy does not necessarily indicate that lifestyle behaviors are unimportant. The relatively modest sample size, predominantly female composition, single-university setting, broad behavioral categories, and reliance on self-report may have limited the ability to detect associations. In addition, BMI is multifactorial and reflects the combined influence of dietary intake, physical activity, sedentary behavior, sleep, genetics, socioeconomic conditions, medications, and environmental factors. Individual behaviors may therefore provide only a partial explanation of variation in BMI.

The findings should also be interpreted in the context of the broader Gulf and global obesity burden. Studies from Saudi Arabia, Oman, Kuwait, and Qatar consistently demonstrate that excess weight is prevalent among young populations, although reported estimates vary (17–21). Globally, the increasing prevalence of overweight and obesity similarly reflects interacting behavioral, environmental, and structural determinants rather than a single lifestyle factor.

### 4.7 Strengths and limitations

This study has several strengths. It examined multiple behavioral domains within the same student population and used both continuous and categorical BMI outcomes. The consistency of the sex and age findings across the adjusted linear and logistic models strengthens the evidence that these demographic characteristics were important correlates of BMI in this sample. The use of robust standard errors in the linear regression also reduced sensitivity to heteroscedasticity.

Several limitations should nevertheless be considered. First, the cross-sectional design prevents conclusions about causality or temporality. Second, the study was conducted at a single university, and the sample was predominantly female and disproportionately represented students from health-related colleges, which limits generalizability. Third, behavioral variables were self-reported and may have been affected by recall and social-desirability bias.

Fourth, dietary assessment was based on selected frequency questions and did not measure portion sizes, total energy intake, or comprehensive dietary quality. Fifth, study did not include complete item-level information necessary to calculate validated PSQI or IPAQ scores. Sixth, several response categories contained very small numbers of participants, particularly for bedtime electronic-device use and some meal-frequency categories. Although Monte Carlo procedures were used for sparse categorical analyses, estimates from these cells remain imprecise.

Seventh, numerous associations were examined, increasing the possibility of chance findings from multiple testing. The statistically significant association between electronic-device use and BMI should therefore be interpreted particularly cautiously. Finally, BMI is an imperfect proxy for adiposity because it does not distinguish fat mass from lean mass or capture fat distribution. Causal associations cannot be determined.

### 4.8 Implications for future research

Future research should address these limitations through larger, multicenter studies involving universities from different regions of Saudi Arabia. Sampling should aim to improve representation across sex, academic discipline, and other relevant demographic characteristics.

Longitudinal studies would be particularly valuable for determining whether BMI changes during university and whether changes in dietary intake, physical activity, sedentary behavior, and sleep precede changes in BMI. Future studies should also use validated and comprehensive instruments. Dietary assessment should incorporate portion sizes, total energy intake, and overall dietary patterns; physical activity should be measured according to frequency, duration, and intensity; sedentary behavior and screen time should be quantified separately; and sleep should preferably be assessed using validated instruments such as the PSQI.

In addition, future research should consider anthropometric measures beyond BMI, including waist circumference or other indicators of central adiposity. Such measures may provide additional information about metabolic risk that cannot be captured by BMI alone.

## 5 Conclusion

In this sample of Saudi university students, 37.2% of participants were classified as overweight or obese. Male sex and older age were the most consistent correlates of higher BMI and overweight/obesity, whereas most individual dietary, physical activity, and sleep behaviors were not independently associated with BMI after adjustment.

The prevalence observed is broadly consistent with the substantial burden of excess weight reported among university students in Saudi Arabia and other Gulf countries (1–5, 8) and occurs within the context of a rapidly increasing global burden of overweight and obesity (6). The consistency of the sex and age associations across analytical approaches suggests that demographic characteristics may be important correlates of weight status in this population. In this single-university cross-sectional sample, overweight and obesity were common. Some dietary measures showed imprecise associations with BMI, while evidence concerning physical activity and sleep was inconclusive. The self-reported measurements, limited sample, and cross-sectional design preclude causal interpretation.

Future multicenter longitudinal studies using validated measures of dietary intake, physical activity, sedentary behavior, and sleep are needed to clarify the determinants and trajectories of overweight and obesity among Saudi university students. Such research could provide a stronger evidence base for culturally appropriate university health strategies aimed at addressing the growing burden of excess weight among young adults.

## Data Availability

The raw data supporting the conclusions of this article will be made available by the authors, without undue reservation.
